# Supplementary material for: From sequence to enzyme mechanism using multi-label machine learning
Source: BMC Bioinformatics. 2014 May 19;15:150. doi: 10.1186/1471-2105-15-150 (PMC4229970; doi:10.1186/1471-2105-15-150)
Supplement: Additional file 2 — Java code of ml2db. Additional file ml2db_code.tar.gz contains the Java source code to run the multi-label machine learning experiments and save the results to database. The code’s Javadoc is included. [file 1471-2105-15-150-S2.zip › additional file 2/ml2db/ml2db/doc/main/ml2db/mulan/mulanarff/database/MulanArffRecord.html]

MulanArffRecord


JavaScript is disabled on your browser.


- Overview
- Package
- Class
- Use
- Tree
- Deprecated
- Index
- Help

- Prev Class
- Next Class

- Frames
- No Frames

- All Classes

- Summary:
- Nested |
- Field |
- Constr |
- Method

- Detail:
- Field |
- Constr |
- Method


main.ml2db.mulan.mulanarff.database

## Class MulanArffRecord

- java.lang.Object
- - uk.ac.standrews.utils.main.Initialised
  - - main.ml2db.mulan.mulanarff.database.MulanArffRecord

- All Implemented Interfaces:
  :   uk.ac.standrews.utils.main.Initialisable

  ---

    

  ```
  public class MulanArffRecord
  extends uk.ac.standrews.utils.main.Initialised
  ```

  Checks that an ARFF record read from the database contains valid parameters
  to read an ARFF file.

  Author:
  :   ldf(at)st-andrews.ac.uk

- - ### Field Summary

    - ### Fields inherited from class uk.ac.standrews.utils.main.Initialised

      `m_initialisationIsCorrect`
  - ### Constructor Summary

    Constructors

    Constructor and Description

    `MulanArffRecord(int arffRecordId,
    DbManagers dbMan)`
  - ### Method Summary

    Methods

    | Modifier and Type | Method and Description |
    | --- | --- |
    | `static java.lang.String` | `checkAndGetFilePath(ArffRecordsTable reader, java.lang.String pathProperty, int arffId)` Returns the file path if the file is readable. |
    | `java.lang.String` | `getArffDbConnPath()` |
    | `java.lang.String` | `getArffFileName()` |
    | `java.lang.String` | `getArffFilePath()` |
    | `int` | `getArffId()` |
    | `uk.ac.standrews.utils.main.database.DbManager` | `getArffRecordDbMan()` |
    | `DbManagers` | `getDbManagers()` |
    | `java.util.Vector<java.lang.String>` | `getInstancesNamesFromFile()` Gets the instances names directly from the comment line above each instance |
    | `static java.util.Vector<java.lang.String>` | `getInstancesNamesFromFile(java.lang.String arffFilePath)` Gets the instances names directly from the comment line above each instance in the ARFF file (eg. |
    | `ArffRecordsTable` | `getReader()` |
    | `java.lang.String` | `getXmlFileName()` |
    | `static void` | `main(java.lang.String[] args)` |

    - ### Methods inherited from class uk.ac.standrews.utils.main.Initialised

      `initialisationIsCorrect, setInitialisation, setInitialisationIsCorrect, setInitialisationNotCorrect`
    - ### Methods inherited from class java.lang.Object

      `equals, getClass, hashCode, notify, notifyAll, toString, wait, wait, wait`

- - ### Constructor Detail


    - #### MulanArffRecord

      ```
      public MulanArffRecord(int arffRecordId,
                     DbManagers dbMan)
      ```
  - ### Method Detail


    - #### checkAndGetFilePath

      ```
      public static java.lang.String checkAndGetFilePath(ArffRecordsTable reader,
                                         java.lang.String pathProperty,
                                         int arffId)
      ```

      Returns the file path if the file is readable.

      Parameters:
      :   `reader` -
      :   `pathProperty` -

      Returns:
      :   the file path if the file is readable, null otherwise


    - #### getArffDbConnPath

      ```
      public java.lang.String getArffDbConnPath()
      ```


    - #### getArffFileName

      ```
      public java.lang.String getArffFileName()
      ```


    - #### getArffFilePath

      ```
      public java.lang.String getArffFilePath()
      ```


    - #### getArffId

      ```
      public int getArffId()
      ```


    - #### getArffRecordDbMan

      ```
      public uk.ac.standrews.utils.main.database.DbManager getArffRecordDbMan()
      ```


    - #### getDbManagers

      ```
      public DbManagers getDbManagers()
      ```


    - #### getInstancesNamesFromFile

      ```
      public java.util.Vector<java.lang.String> getInstancesNamesFromFile()
      ```

      Gets the instances names directly from the comment line above each
      instance

      Returns:


    - #### getReader

      ```
      public ArffRecordsTable getReader()
      ```


    - #### getXmlFileName

      ```
      public java.lang.String getXmlFileName()
      ```


    - #### getInstancesNamesFromFile

      ```
      public static java.util.Vector<java.lang.String> getInstancesNamesFromFile(java.lang.String arffFilePath)
      ```

      Gets the instances names directly from the comment line above each
      instance in the ARFF file (eg. % instance\_1 )

      Returns:
      :   the instances names


    - #### main

      ```
      public static void main(java.lang.String[] args)
      ```


- Overview
- Package
- Class
- Use
- Tree
- Deprecated
- Index
- Help

- Prev Class
- Next Class

- Frames
- No Frames

- All Classes

- Summary:
- Nested |
- Field |
- Constr |
- Method

- Detail:
- Field |
- Constr |
- Method
